# Involvement of RSK1 activation in malformin-enhanced cellular fibrinolytic activity

**DOI:** 10.1038/s41598-018-23745-0

**Published:** 2018-04-03

**Authors:** Yukio Koizumi, Kenichiro Nagai, Lina Gao, Souichi Koyota, Tomokazu Yamaguchi, Miyuki Natsui, Yumiko Imai, Keiji Hasumi, Toshihiro Sugiyama, Keiji Kuba

**Affiliations:** 1 Department of Biochemistry and Metabolic Science, Akita University Graduate School of Medicine, 1-1-1 Hondo, Akita, 010-8543 Japan; 2 School of Pharmacy, Kitasato University, 5-9-1 Shirokane, Minato-ku Tokyo, 108-8641 Japan; 3 Molecular Medicine Laboratory, Bioscience Education and Research Support Center, Akita University, 1-1-1 Hondo, Akita, 010-8543 Japan; 4 Laboratory of Regulation of Intractable Infectious Diseases, National Institute of Biomedical Innovation, Health and Nutrition, 7-6-8 Saito-Asagi, Ibaraki, Osaka, 567-0085 Japan; 5 Department of Applied Biological Science, Tokyo Noko University, 3-5-8 Saiwaicho, Fuchu Tokyo, 183-8509 Japan

## Abstract

Pharmacological interventions to enhance fibrinolysis are effective for treating thrombotic disorders. Utilizing the *in vitro* U937 cell line-based fibrin degradation assay, we had previously found a cyclic pentapeptide malformin A_1_ (MA_1_) as a novel activating compound for cellular fibrinolytic activity. The mechanism by which MA_1_ enhances cellular fibrinolytic activity remains unknown. In the present study, we show that RSK1 is a crucial mediator of MA_1_-induced cellular fibrinolysis. Treatment with rhodamine-conjugated MA_1_ showed that MA_1_ localizes mainly in the cytoplasm of U937 cells. Screening with an antibody macroarray revealed that MA_1_ induces the phosphorylation of RSK1 at Ser380 in U937 cells. SL0101, an inhibitor of RSK, inhibited MA_1_-induced fibrinolytic activity, and CRISPR/Cas9-mediated knockout of RSK1 but not RSK2 suppressed MA_1_-enhanced fibrinolysis in U937 cells. Synthetic active MA_1_ derivatives also induced the phosphorylation of RSK1. Furthermore, MA_1_ treatment stimulated phosphorylation of ERK1/2 and MEK1/2. PD98059, an inhibitor of MEK1/2, inhibited MA_1_-induced phosphorylation of RSK1 and ERK1/2, indicating that MA_1_ induces the activation of the MEK-ERK-RSK pathway. Moreover, MA_1_ upregulated the expression of urokinase-type plasminogen activator (uPA) and increased uPA secretion. These inductions were abrogated in RSK1 knockout cells. These results indicate that RSK1 is a key regulator of MA_1_-induced extracellular fibrinolytic activity.

## Introduction

Irregular thrombus formation causes severe ischemic diseases such as cerebral infarction and myocardial infarction. Antithrombotic therapies are categorized into three groups: anticoagulants, antiplatelet agents, and thrombolytics. A thrombolytic therapy induces the conversion of proenzyme plasminogen to plasmin, a fibrinolytic enzyme, which in turn leads to the degradation of insoluble fibrin, the main component of thrombus. Although thrombolytic enzymes, such as tissue-type plasminogen activator (tPA) or urokinase-type plasminogen activator (uPA; also known as urokinase), are available for the treatment of thrombotic disorders, pharmacological interventions using low molecular weight compounds for thrombolytic therapies currently present an unmet medical need.

**Figure 1:**
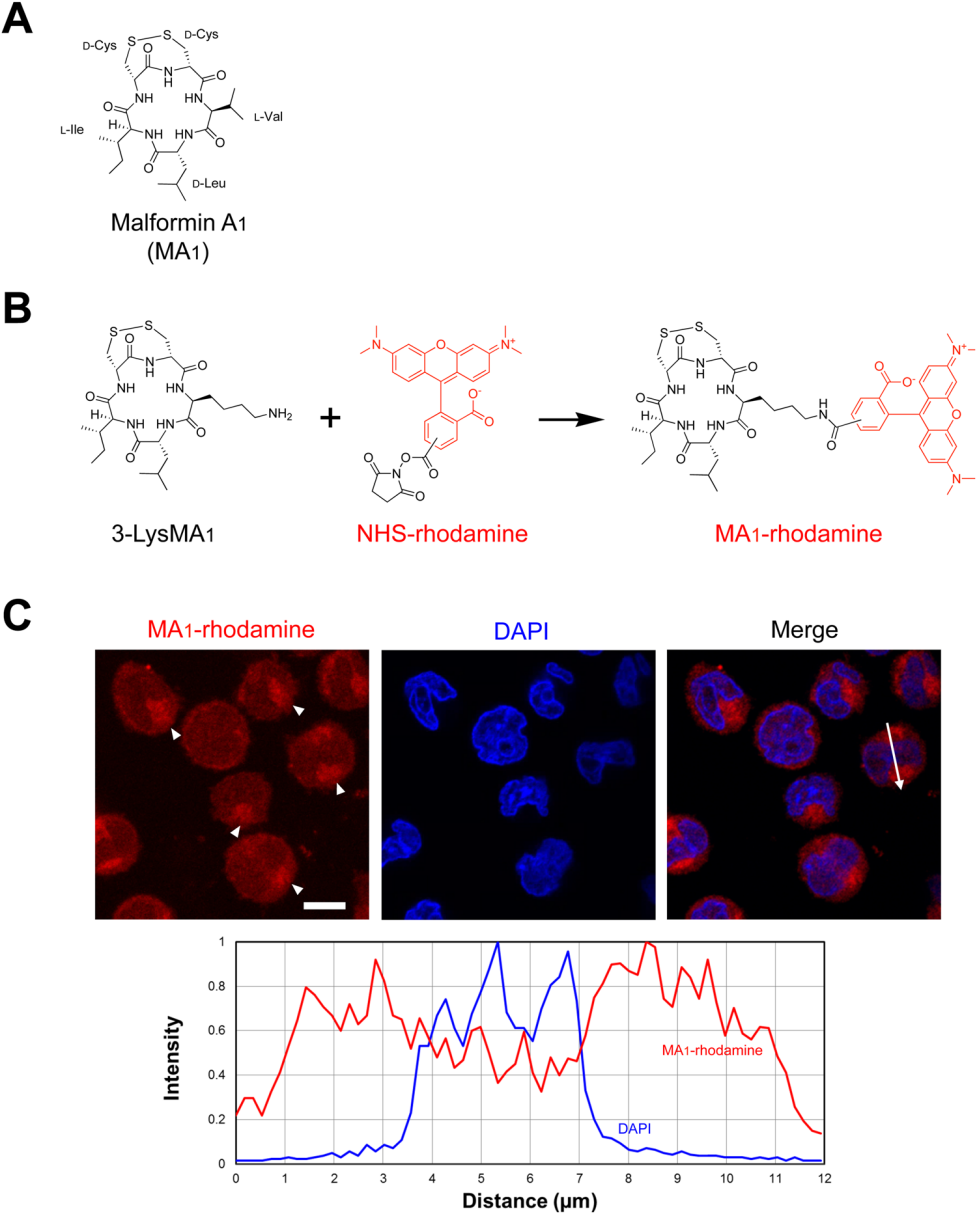
Fluorescence-conjugated MA_1_ is localized to the intracellular compartment. (**A**) Structure of MA_1_. (**B**) Strategy for MA_1_-rhodamine synthesis. (**C**) MA_1_-rhodamine localization in U937 cells. MA_1_-rhodamine-treated cells were counter-stained with DAPI and analyzed with a confocal laser scanning microscope. MA_1_-rhodamine staining (red) (*top left*), DAPI staining (blue) (*top center*), and merged image (*top right*) are shown. The histogram at the bottom indicates localization of MA_1_-rhodamine and DAPI signal intensities following the line in the merged image (*top right*). Scale bar, 10 µm. Arrowheads, MA_1_-rhodamine-enriched regions.

By utilizing an *in vitro* U937 monocytoid cell line-based fibrin degradation assay, we had previously found the natural cyclic pentapeptide malformin A_1_ (MA_1_), a disulfide form of *cyclo*(-D-Cys-D-Cys-L-Val-D-Leu-L-Ile-), as a novel activating compound for cellular fibrinolytic activity (Fig. 1A)^1^. MA_1_ was originally identified as a fungal metabolite that induces curvatures on bean plants and on corn roots^2–5^. Thereafter, the various biological activities of MA_1_ and its analogs have been reported^6–18^. The mode of action of MA_1_ in enhancing the fibrinolytic activity involves the uPA/plasminogen system on the cell surface and depends on the coordinated action of vitronectin as a plasma component^1,19^. However, the intracellular mechanism by which MA_1_ enhances fibrinolytic activity remains unclear^19^.

The mitogen-activated protein kinase (MAPK) pathway has been shown to regulate multiple cellular processes including cell proliferation, differentiation, and survival^20^. The MAPK pathway transduces extracellular signals to intracellular target proteins through the recruitment and activation of various signaling molecules such as Ras, Raf, MAPK kinase (MEK), and extracellular signal-regulated kinase (ERK). Once activated, ERK phosphorylates several substrates, including the p90 ribosomal S6 kinase (RSK)^21^. RSK activation is regulated by sequential phosphorylations controlled by upstream activators, including ERK1/2 and PDK1, as well as by autophosphorylation^22–27^.

In this study, we investigated mechanisms of action by which MA_1_ enhances cellular fibrinolytic activity. An antibody-spotted macroarray screening revealed that MA_1_ treatment induces the phosphorylation of RSK1 at Ser380. Pharmacological and genetic intervention of RSK inhibited MA_1_-enhanced fibrinolytic activity. Moreover, MA_1_ treatment caused the activation of the MEK-ERK-RSK pathway and increased the expression of uPA. These results show that RSK1 is a key regulator of MA_1_-enhanced cellular fibrinolytic activity.

## Results

### Fluorescence-conjugated MA_1_ is localized to the intracellular compartment

In our previous report, MA_1_-pretreated U937 cells exerted significant pro-fibrinolytic activities, suggesting that a MA_1_ target was present in the cellular fractions^1^. We evaluated the cellular distribution of MA_1_ using a synthesized fluorescence probe (Fig. 1B). Rhodamine-conjugated MA_1_ was observed mainly in the cytoplasm of U937 cells (Fig. 1C and Fig. S1), and enrichment of MA_1_-rhodamine was also observed in cytoplasmic organelles in some of the cells (Fig. 1C). These results suggest that MA_1_ may interact with intracellular molecule(s).

### Antibody macroarray screening reveals that MA_1_ induces phosphorylation of RSK1

**Figure 2:**
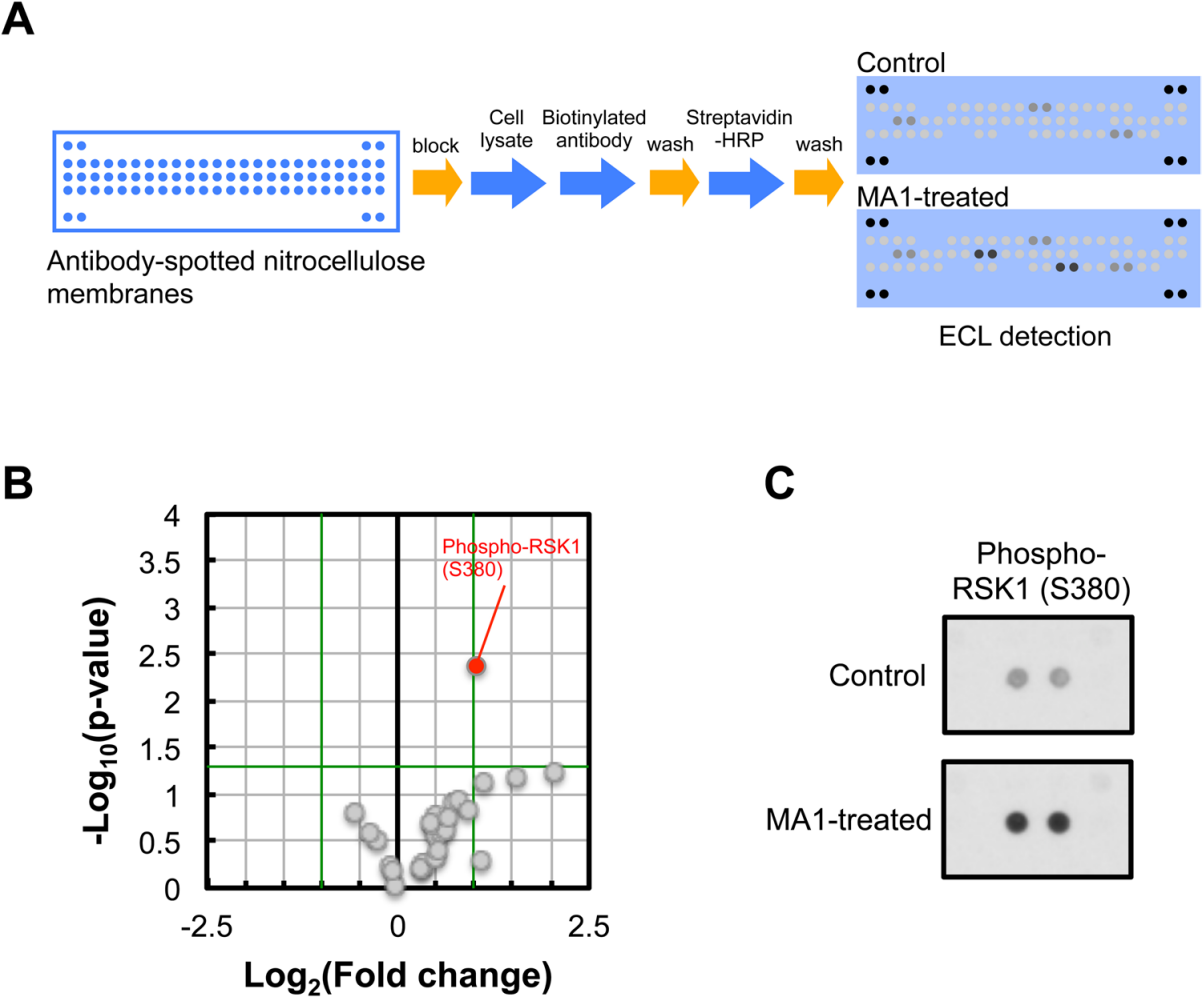
Membrane-based antibody macroarray. (**A**) Procedure for membrane-based antibody macroarray. Macroarray membranes were blocked with blocking buffer and then simultaneously reacted with cell lysates and a biotinylated antibody cocktail. After washing, the membranes were incubated with streptavidin-conjugated HRP. Subsequently, multiple proteins were detected using the ECL system. (**B**) Volcano plot of antibody macroarray data created by quantifying the mean spot pixel densities for MA_1_-treated versus non-treated control U937 cell lysate. The x-axis is the fold change between the two samples represented as log_2_([MA_1_]/[Control]) and the y-axis is the significance between the two samples represented as −log_10_ (p-value). Statistical analyses were performed using the Student’s t test. Green lines show cut-off values (fold change >2, p-value <0.05). The macroarray was conducted with n = 3. (**C**) Macroarray images showing the spots of phospho-RSK1 (Ser380). Upper; non-treated control, lower; MA_1_-treated.

To address what intracellular mechanisms by which MA_1_ induces fibrinolytic activity, we carried out a screening to identify differentially phosphorylated kinases (26 targets) between MA_1_-treated and non-treated cell lysates using membrane-based antibody macroarrays (Fig. 2A). The result was represented as a Volcano plot (Fig. 2B). The negative log_10_ function of the p-value was plotted against the log_2_ function of the fold change, representing the up- and downregulated phosphorylated kinases in response to MA_1_ treatment. All kinases with increasing or decreasing fold changes by at least 2-fold at a p-value cut-off of p < 0.05 were considered differentially phosphorylated as a result of the treatment. MA_1_ significantly elevated the signal intensity of phosphorylated RSK1 (Ser380) (Fig. 2B,C, Table S1).

### MA_1_-induced RSK1 phosphorylation is independent of fibrin and plasma components

**Figure 3:**
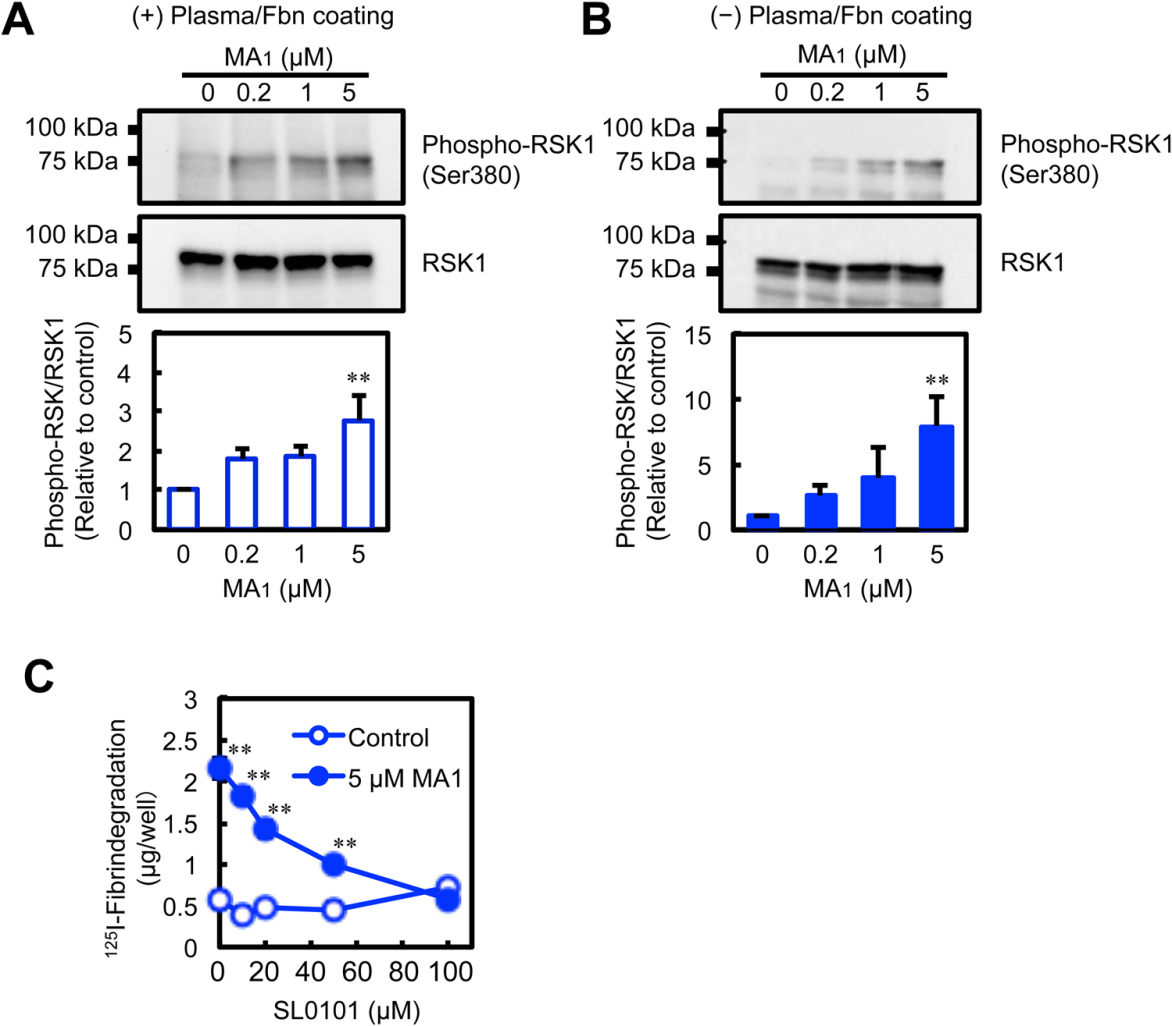
RSK1 is involved in MA_1_-enhanced fibrinolytic activity. (**A**,**B**) Western blot analysis of phospho-RSK1 (Ser380) and RSK1 in MA_1_-treated U937 cells. MA_1_ treatment was performed in the presence of human platelet-poor plasma on a fibrin-coated dish (**A**) or in the absence of plasma on a non-coated dish (**B**) at 37 °C for 1 h. Data were presented as means ± SD (n = 3). Statistical analyses were performed using one-way ANOVA with Tukey’s post-hoc test (^**^p < 0.01, versus control). (**C**) Effect of SL0101 on MA_1_-enhanced fibrinolytic activity. For the *in vitro* fibrin degradation assay, U937 cells with human platelet-poor plasma were added to each well of the ^125^I-fibrin-coated 96-well microplate. After incubation for 3 h with MA_1_ and SL0101 at the indicated concentrations, ^125^I-fibrin degradation products released into the supernatant were quantified using a γ-counter. Data were presented as means ± SD (n = 3). Statistical analyses were performed using two-way ANOVA with Tukey’s post-hoc test (^**^p < 0.01, versus control).

We further examined the phosphorylation status of RSK1 by western blotting. Phosphorylation of Ser380 residue of RSK1 was increased by MA_1_ in U937 cells cultured in the presence of plasma components on the fibrin-coated plate (Fig. 3A). As we had previously shown that the uPA/plasminogen system and vitronectin were involved in MA_1_-induced fibrinolysis^19^, we next examined whether extracellular components affected the phosphorylation status of RSK1. When we cultured U937 cells in the absence of plasma components on non-coated plates, MA_1_ also stimulated phosphorylation of RSK1 (Fig. 3B). These data show that fibrin and/or plasma components are not necessary for the ability of MA_1_ to stimulate RSK1 phosphorylation in U937 cells.

### RSK1 is essential for MA_1_-enhanced fibrinolytic activity

**Figure 4:**
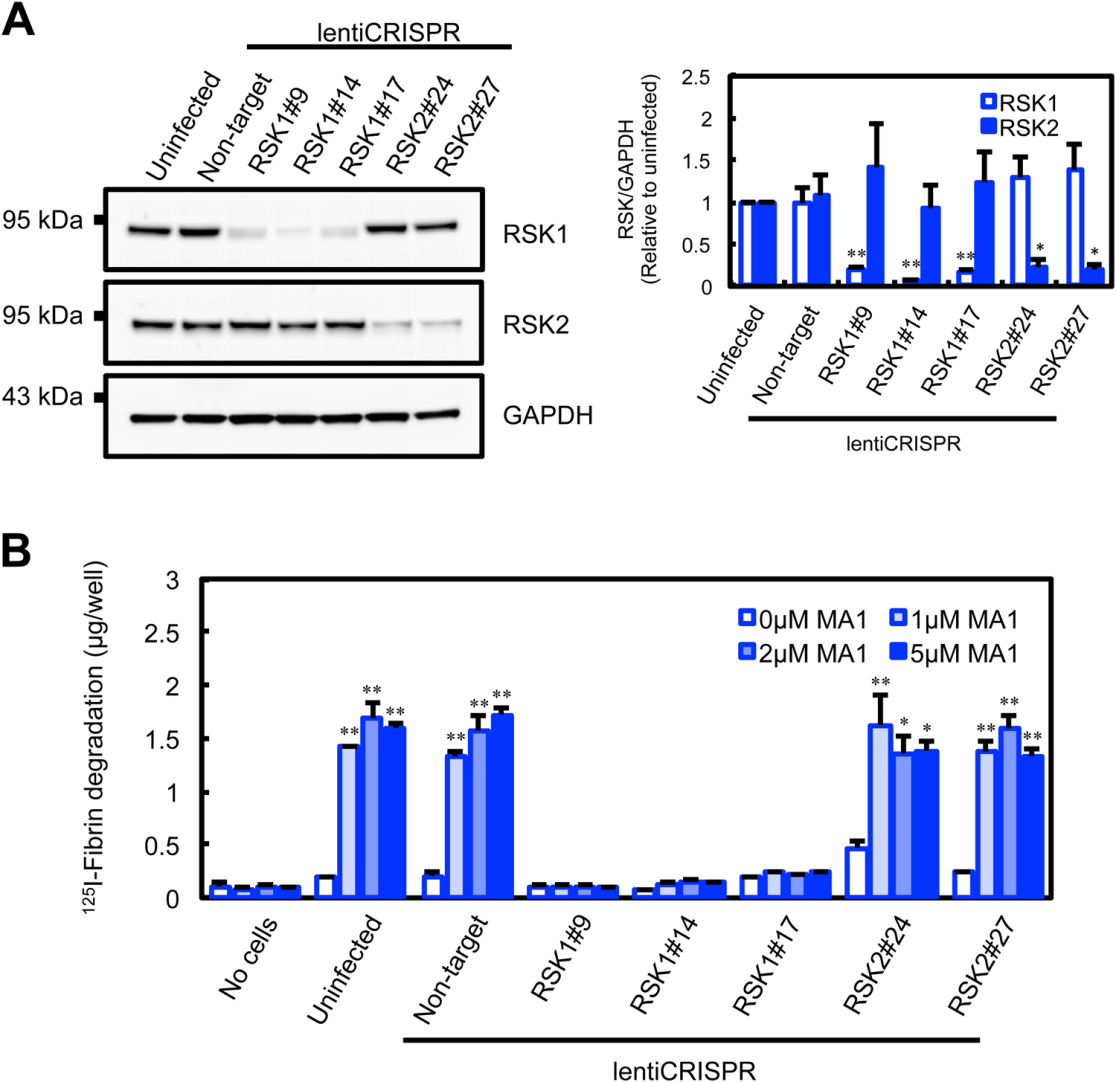
CRISPR/Cas9-mediated RSK1 knockout suppresses MA_1_-enhanced fibrinolytic activity. (**A**) Western blot analysis of RSK1 and RSK2 in CRISPR/Cas9-mediated knockout (KO) U937 cell clones, RSK1#9, RSK1#14, RSK1#17, RSK2#24 and RSK2#27. (**B**) Effect of MA_1_ on fibrinolytic activity in CRISPR/Cas9-mediated KO U937 cell clones. CRISPR/Cas9-mediated KO U937 cell clones with human platelet-poor plasma were added to each well of the ^125^I-fibrin-coated 96-well microplate. After incubation for 3 h with MA_1_ at the indicated concentrations, ^125^I-fibrin degradation products released into the supernatant were quantified using a γ-counter. Data were presented as means ± SD (n = 3). Statistical analyses were performed using one-way ANOVA with Tukey’s post-hoc test (^*^p < 0.05, ^**^p < 0.01, versus control).

The phosphorylation of RSK1 at several sites including Ser380 is important for its kinase activation and downstream signaling^22–27^. To address whether RSK1 activation was involved in MA_1_-enhanced cellular fibrinolytic activity, we co-treated U937 cells with an RSK inhibitor and MA_1_ on fibrin-coated plates. SL0101, an inhibitor of RSK^28^, suppressed MA_1_-enhanced fibrinolytic activity in a dose-dependent manner (Fig. 3C). We next analyzed the effects of RSK1 gene knockout (KO) on MA_1_-enhanced fibrinolysis via genome editing with the CRISPR/Cas9 system. U937 cells transduced with lentivirus co-expressing Cas9 nuclease with RSK1- or RSK2-targeted sgRNA, or non-targeted control sgRNA were collected after puromycin selection, and single cell clones with a deletion of RSK1 (RSK1#9, RSK1#14, and RSK1#17) or RSK2 (RSK2#24 and RSK2#27) were further selected. Protein expression of RSK1 was depleted in the cell clones RSK1#9, RSK1#14, and RSK1#17; whereas RSK2 expression was depleted in the clones RSK2#24 and RSK2#27 (Fig. 4A). In non-target sgRNA-expressing cells and RSK2 KO cells, MA_1_ induced fibrinolytic activity (Fig. 4B). By contrast, MA_1_ failed to enhance fibrinolytic activity in RSK1 KO clones (Fig. 4B). These data indicate that RSK1 is functionally involved in MA_1_-enhanced cellular fibrinolytic activity.

### Biologically active MA_1_ derivative also induces phosphorylation of RSK1

**Figure 5:**
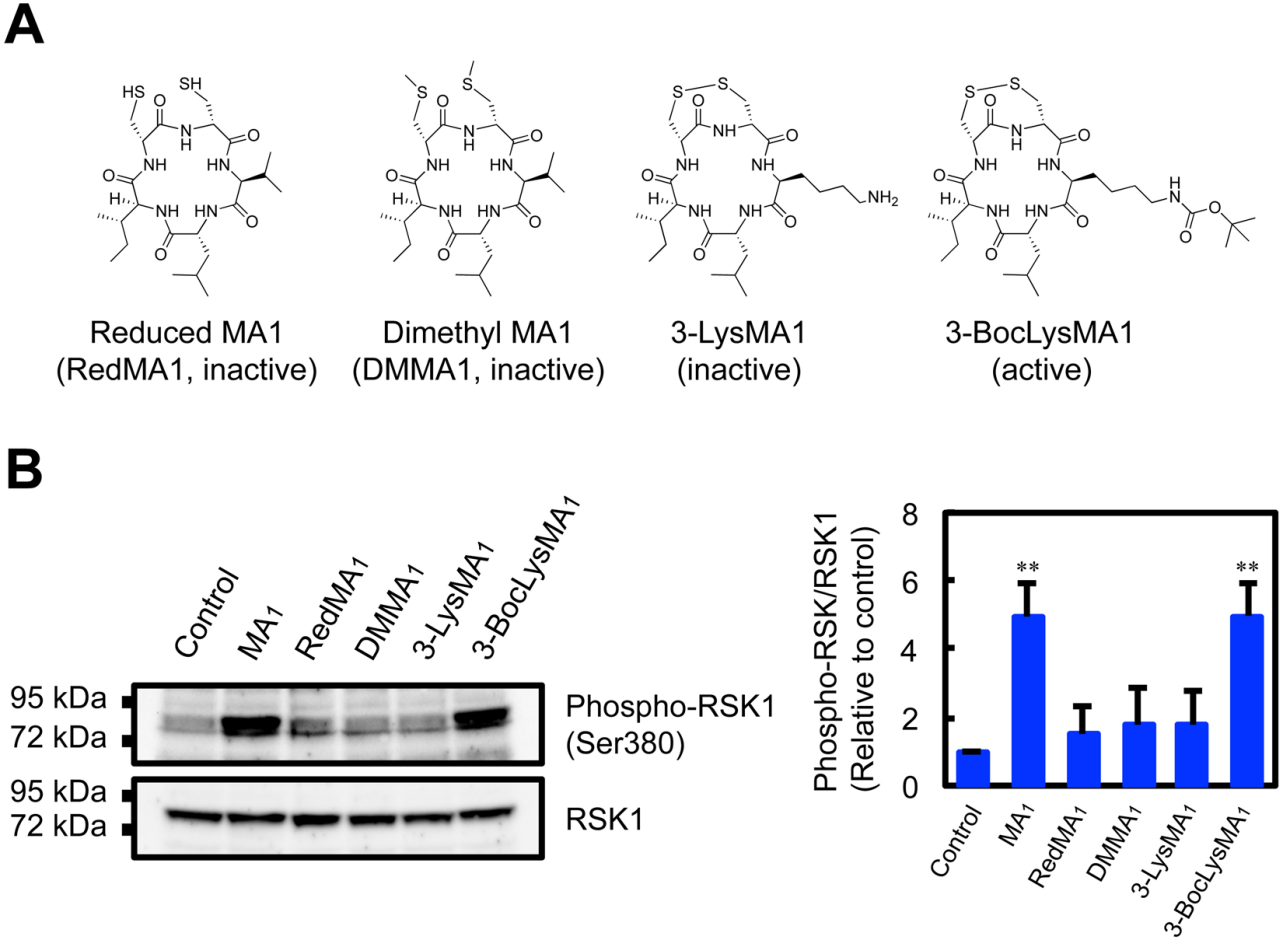
Biologically active MA_1_ derivative induces phosphorylation of RSK1. (**A**) Structures of reduced MA_1_, dimethyl MA_1_, 3-LysMA_1_, and 3-BocLysMA_1_. 3-BocLysMA_1_ is biologically active, and the other three derivatives are not. (**B**) Western blot analysis of phospho-RSK1 (Ser380) and RSK1 in MA_1_ derivative-treated U937 cells. Treatment with 5 µM MA_1_ derivatives was performed at 37 °C for 1 h. RedMA_1_; reduced MA_1_, DMMA_1_; dimethyl MA_1_. Data were presented as means ± SD (n = 3). Statistical analyses were performed using one-way ANOVA with Tukey’s post-hoc test (^**^p < 0.01, versus control).

Our previous structure-activity relationship study showed that the disulfide bond and the bulky hydrophobic side chains in MA_1_ play a crucial role in exerting pro-fibrinolytic activity^29^. We further examined the effects of synthetic MA_1_ derivatives on RSK1 phosphorylation. Three MA_1_ derivatives (reduced MA_1_, dimethyl MA_1_, and 3-LysMA_1_), which have been found not to retain fibrinolysis-enhancing activity, did not induce phosphorylation of RSK1 (Fig. 5A,B). On the other hand, a biologically active derivative, 3-BocLysMA_1_, induced RSK1 phosphorylation (Fig. 5A,B). Thus, there was a positive correlation between the biological activity and the induction of RSK1 phosphorylation in MA_1_ derivatives.

### MA_1_ induces the activation of the MEK-ERK-RSK pathway

**Figure 6:**
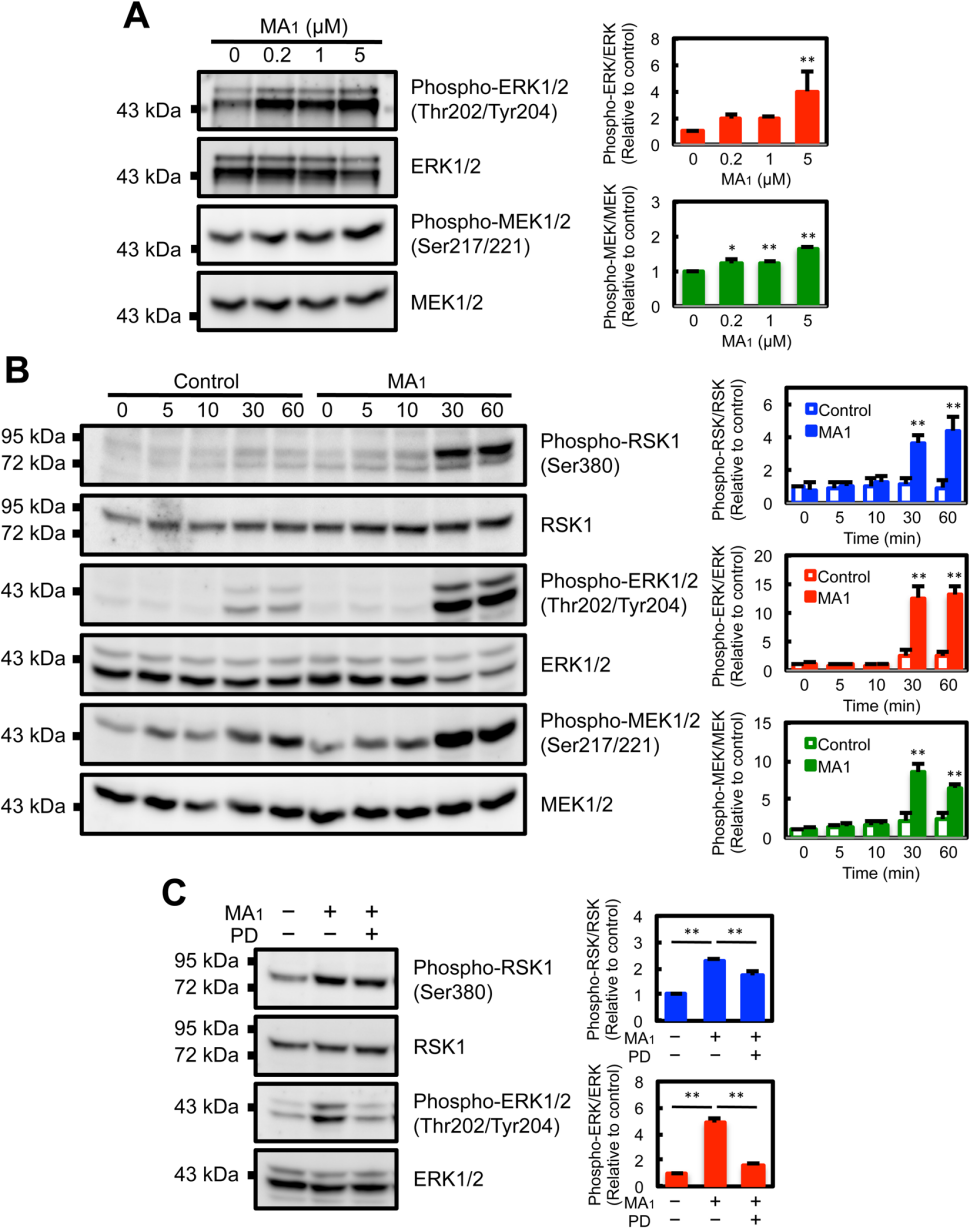
MA_1_ induces the phosphorylation of ERK1/2 and MEK1/2. (**A**) Western blot analysis of phospho-ERK1/2 (Thr202/Tyr204) and ERK1/2, and phospho-MEK1/2 (Ser217/Ser221) and MEK1/2 in MA_1_-treated U937 cells. MA_1_ treatment was performed at 37 °C for 1 h. Data were presented as means ± SD (n = 3). Statistical analyses were performed using one-way ANOVA with Tukey’s post-hoc test (^*^p < 0.05, ^**^p < 0.01, versus control). (**B**) Western blot time course analysis of phosphorylation of RSK1, ERK1/2, and MEK1/2 in MA_1_-treated U937 cells. U937 cells were treated with 5 μM MA_1_ at 37 °C for the indicated times. Data were presented as means ± SD (n = 3). Statistical analyses were performed using two-way ANOVA with Tukey’s post-hoc test (^**^p < 0.01, versus control). (**C**) Effects of PD98059 on MA_1_-induced RSK1 and ERK1/2 phosphorylation. U937 cells were treated with 5 μM MA_1_ and 100 µM PD98059 at 37 °C for 1 h. Data were presented as means ± SD (n = 3). Statistical analyses were performed using one-way ANOVA with Tukey’s post-hoc test (^**^p < 0.01, versus MA_1_-only treatment).

RSK1 is activated by MAP kinases of the ERK family in response to growth factors, hormones, and other stimuli^21^. We investigated whether ERK1/2, an upstream kinase of RSK1, was activated in MA_1_-treated cells. MA_1_ treatment caused the phosphorylation of ERK1/2 (Fig. 6A). Furthermore, MA_1_ also upregulated phosphorylation levels of MEK1/2, an upstream kinase of ERK1/2 (Fig. 6A). These inductions were observed identically to RSK1 phosphorylation at 30 min after MA_1_ stimulation (Fig. 6B). MA_1_-induced activation of RSK1 and ERK1/2 was suppressed by PD98059 (Fig. 6C), an inhibitor of MEK^30^. These results show that MA_1_ induces the activation of the MEK-ERK-RSK pathway.

### MA_1_ increases the expression of the uPA gene and secretion of uPA in an RSK1-dependent manner

**Figure 7:**
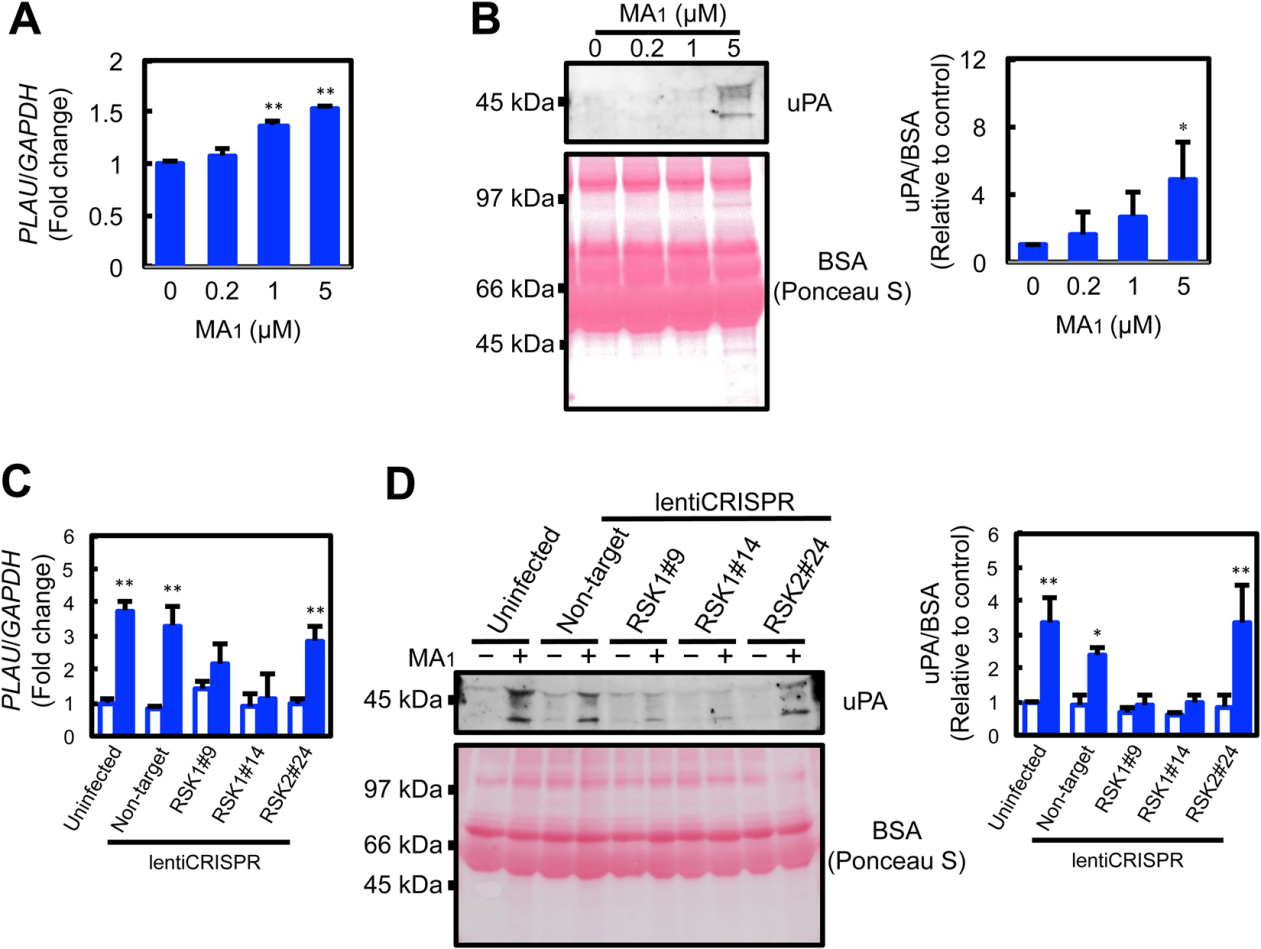
MA_1_ increases the expression of the uPA gene and the secretion of uPA in an RSK1-dependent manner. (**A**) Quantitative real-time PCR for the determination of uPA gene expression levels. U937 cells were treated with MA_1_ at the indicated concentration at 37 °C for 1 h. RNA was then extracted and the expression of the uPA gene (*PLAU*) was quantified by qPCR. (**B**) Western blot analysis of uPA in the conditioned medium. U937 cells were treated with MA_1_ at the indicated concentration at 37 °C for 3 h. Conditioned media were concentrated ~20-fold and subjected to western blot analysis for the detection of uPA. Data were presented as means ± SD (n = 3). Statistical analyses were performed using one-way ANOVA with Tukey’s post-hoc test (^*^p < 0.05, ^**^p < 0.01, versus control). (**C**) Quantitative real-time PCR for the determination of uPA gene expression levels in CRISPR/Cas9-mediated U937 knockout clones. Knockout clones (RSK1#9, RSK1#14, or RSK2#24) were treated with 5 μM MA_1_ at 37 °C for 1 h. RNA was then extracted and the expressions of uPA gene (*PLAU*) were quantified by qPCR. (**D**) Western blot analysis of uPA in the conditioned medium of CRISPR/Cas9-mediated U937 knockout clones. Knockout clones (RSK1#9, RSK1#14, or RSK2#24) were treated with 5 μM MA_1_ at 37 °C for 3 h. Conditioned media were concentrated ~20-fold and subjected to western blot analysis for the detection of uPA. Data were presented as means ± SD (n = 3). Statistical analyses were performed using two-way ANOVA with Tukey’s post-hoc test (^*^p < 0.05, ^**^p < 0.01, versus control).

**Figure 8:**
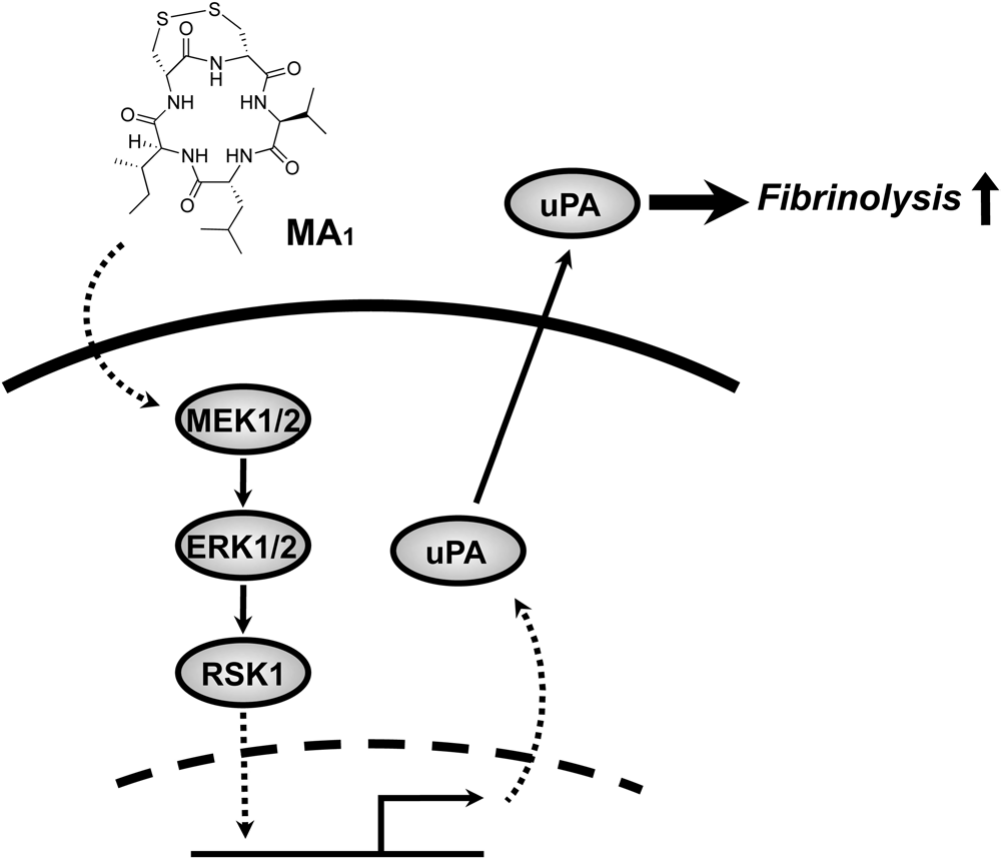
A proposed model of MA_1_-mediated MEK-ERK-RSK1 pathway activation and subsequent expression of uPA followed by fibrinolysis enhancement.

Although MA_1_-induced RSK activation increased fibrinolytic activity, it remains unknown what fibrinolytic factors were elevated. Our previous study using neutralizing antibodies has shown that uPA was involved in MA_1_-enhanced activity^1^. Therefore, we examined uPA gene expression levels. The expression of the uPA gene was upregulated in MA_1_-treated cells (Fig. 7A). Furthermore, when the uPA protein level was examined, MA_1_ induced an increase in uPA secretion into the conditioned medium (Fig. 7B). Next, we investigated the effects of RSK1 KO. In contrast to the RSK2 KO clone (RSK2#24), MA_1_ did not significantly upregulate the expression of the uPA gene in RSK1 KO clones (RSK1#9 and RSK1#14) (Fig. 7C). Similarly, MA_1_ could not increase the secretion of uPA in RSK1 KO clones (Fig. 7D). These results indicate that MA_1_ induced the activation of RSK1 followed by an increase in uPA expression, and enhanced extracellular fibrinolytic activity (Fig. 8).

## Discussion

In our previous report, we had shown that MA_1_-treated U937 cells exhibited changes in actin organization and focal accumulation of plasminogen on the cell surface^19^. In the present study, the fluorescence-labeled MA_1_, when added to the culture of U937 cells, was primarily localized to the intracellular compartment (Fig. 1C). Based on these observations, we hypothesized that MA_1_ may affect the function of intracellular molecules. Indeed, we showed here that RSK1 was essentially activated by MA_1_ and thereby enhanced fibrinolytic activity. Activation of intracellular signaling by MA_1_ had also been previously reported to have other effects including cytotoxic effects of MA_1_ on prostate cancer cells involving the accumulation of reactive oxygen species, a decrease in mitochondrial transmembrane potential, and induction of autophagy^10^. Importantly, MA_1_ induced the activation of ERK, an upstream kinase of RSK, and its activation was considered to occur in a MEK-dependent pathway (Fig. 6). Localization at the intracellular compartment of MA_1_ may be necessary for efficient activation of the MEK-ERK-RSK pathway, which increases cellular fibrinolytic activity. The identification of direct target molecules of MA_1_ that activates MEK is currently under investigation.

The RSK family is comprised of a group of highly related serine/threonine kinases that regulate diverse cellular processes, including cell growth, proliferation, survival, and motility. This family includes four vertebrate isoforms (RSK1–4). Mutations in the RSK2 isoform are known to be associated with Coffin-Lowry syndrome^31^, and a gene knockout study of RSK2 in mice shows that RSK2 is a modulator of craniofacial development^32^. Results from these previous reports plus the results of this study that RSK1 KO specifically suppresses MA_1_-induced fibrinolytic activity (Fig. 4B, and Fig. 7C,D) lead us to speculate that RSK family proteins are exhibit isoform-specific functions. As the phenotypes of RSK1 KO mice have not yet been reported, the significance of RSK1 in fibrinolysis *in vivo* remains elusive.

Mammalian RSK was reported to function as an important effector of ERK in global transcriptional regulation^33^. RSK was shown to regulate ~20% of mRNAs controlled by ERK in Madin-Darby canine kidney cells through direct and indirect mechanisms, such as the induction of transcription factors. On the other hand, there have been several reports describing the regulation of uPA activity via the activation of RSK^33,34^. Activated RSK regulates epithelial cell motility and invasiveness by stimulating the gene expression of the uPA/uPA receptor^33^, and activated RSK in breast carcinoma enhances cellular invasion through phosphorylation of IκBα, allowing NF-κB to translocate into the nucleus and induce uPA expression^34^. We had previously shown that uPA rather than tPA was likely to be involved in MA_1_-enhanced fibrinolytic activity^1^, and in this study, MA_1_ consistently upregulated uPA mRNA levels and increased uPA protein secretion (Fig. 7A,B). Target gene clusters for MA_1_-induced RSK1-mediated transcription activation will be further elucidated in the future.

In summary, we demonstrated that the phosphorylation of RSK1 at Ser380 was crucial for MA_1_-induced cellular fibrinolysis in U937 monocytoid cells. Pharmacological and genetic intervention of RSK1 suppressed MA_1_-enhanced fibrinolytic activity. In addition, MA_1_ resulted in the activation of the MEK-ERK pathway followed by the activation of RSK. Further, MA_1_ led to the induction of uPA expression in an RSK-dependent manner. Although further studies are needed, the potential applications of MA_1_ derivatives or RSK1-activating compounds as therapeutics may contribute to the development of better antithrombotic treatments for severe ischemic diseases.

## Methods

### Reagents

NHS-Rhodamine, Opti-MEM, anti-uPA antibody, and TRIzol reagent were purchased from Thermo Fisher Scientific. DAPI was obtained from Dojindo. Fibrinogen was purchased from Sigma-Aldrich. RSK inhibitor SL0101 and MEK inhibitor PD98059 were obtained from Merck Millipore. Anti-phospho-RSK1 (Ser380), anti-RSK1, anti-RSK2, anti-GAPDH, anti-phospho-ERK1/2 (Thr202/Tyr204), and anti-phospho-MEK1/2 (Ser217/Ser221) antibodies were purchased from Cell Signaling Technology. Anti-ERK1/2, and anti-MEK1/2 antibodies were purchased from Santa Cruz Biotechnology. LentiCRISPRv2, pMD2.G, and psPAX2 were obtained from Addgene. FuGENE HD was purchased from Promega. PrimeScript RT reagent Kit and SYBR Premix Ex Taq II were obtained from TaKaRa.

### Malformin A_1_ and its synthetic derivatives

MA_1_ was purified from the culture broth of *Aspergillus niger* F7586 as previously described^19^. Reduced MA_1_, Dimethyl MA_1_, 3-LysMA_1_, and 3-BocLysMA_1_ were synthesized using solid-phase peptide synthesis according to previous work^29^. MA_1_-rhodamine was synthesized by conjugation of 3-LysMA_1_ with NHS-rhodamine in 20 mM phosphate buffer (pH 7.4) at 37 °C for 2 h, and then purified by preparative HPLC.

### Cell culture

U937 cells were obtained from Japanese Collection of Research Bioresources, and were cultured in RPMI-1640 including 10% FBS, 100 units/mL penicillin, and 100 µg/mL streptomycin. 293FT cells were obtained from Thermo Fisher Scientific, and were cultured in complete medium including DMEM, 10% FBS, 6 mM L-glutamine, 0.1 mM non-essential amino acids, 1 mM sodium pyruvate, 100 units/mL penicillin, and 100 µg/mL streptomycin. Cells were cultured in a humidified 5% CO_2_ atmosphere at 37 °C.

### Fluorescence imaging

For MA_1_-rhodamine staining, U937 cells (5 × 10^6^ cells/ml) were incubated with 5 µM MA_1_-rhodamine in 10% human platelet-poor plasma on fibrin-coated 8-chamber slide at 37 °C for 1 h. After the reaction, cells were fixed with 3.7% paraformaldehyde at room temperature for 20 min. The cells were subsequently washed twice with PBS and incubated with DAPI for 10 min in the dark. Fluorescence images were taken with a confocal laser scanning microscope LSM510 (Carl Zeiss).

### Membrane-based antibody macroarray

Membrane-based antibody macroarrays were performed using Proteome Profiler Human phosphorylated kinase Arrays (ARY002B; R&D Systems). The procedure was carried out according to the manufacturer’s instruction. Briefly, U937 cells (5 × 10^6^ cells/ml) were treated with 5 µM MA1 in 10% human platelet-poor plasma on a fibrin-coated 24-well microplate at 37 °C for 1 h. After treatment, cell lysates were prepared in lysis buffer (contained in the kit) and clarified by centrifugation. After determination of protein concentration, equal amounts of protein were subjected to macroarrays. Macroarray membranes were blocked with blocking buffer (contained in the kit) and then simultaneously reacted with cell lysates and a biotinylated antibody cocktail. After washing, the membranes were incubated with streptavidin-conjugated horseradish peroxidase. Subsequently, multiple phosphorylated kinases were detected using the ECL system (GE Healthcare). The macroarray was conducted with n = 3.

### Western blot analysis

U937 cells (5 × 10^6^ cells/ml) were treated with test compounds at the indicated concentrations in the presence or absence of 10% human platelet-poor plasma on a fibrin-coated or non-coated dish at 37 °C for 1 h. After treatment, cell lysates were prepared in lysis buffer (50 mM Tris-HCl [pH 7.5], 150 mM NaCl, 1 mM EDTA, 1% NP-40, 20 mM NaF, 2 mM Na_3_VO_4_, and protease inhibitor cocktail [Roche Diagnostics]) and clarified by centrifugation. After determining the protein concentration, equal amounts of protein were subjected to SDS-PAGE under reducing conditions. For uPA detection, U937 cells were treated with MA_1_ for 3 h in RPMI-1640 medium containing 0.5% FBS. After treatment, conditioned media were concentrated ~20-fold using Amicon ultra filter devices (Merck Millipore), and then subjected to SDS-PAGE under non-reducing conditions. Next, proteins were transferred to a nitrocellulose membrane, and then blocked with 5% skim milk and then incubated with specific antibodies. After washing, the membranes were incubated with peroxidase-conjugated secondary antibody. Subsequently, targeted proteins were detected using the ECL system.

### *In vitro* fibrin degradation assay

The *In vitro* fibrin degradation assay was performed using an ^125^I-labeled fibrin-coated plate as previously described^1,19^. Briefly, each well of a 96-well microplate was coated with ^125^I-fibrinogen (ca. 50,000 cpm/20 µg/well) and then treated with 0.68 units/ml thrombin to convert ^125^I-fibrinogen to ^125^I-fibrin. Fifty microliters of U937 cells at a concentration of 5 × 10^6^ cells/ml in 10% human platelet-poor plasma were added to each well of the ^125^I-fibrin-coated 96-well microplate. After incubating at 37 °C for 3 h with samples at the indicated concentrations, ^125^I-fibrin degradation products released into the supernatant were quantified using the γ-counter AccuFLEX γ7010 (Hitachi).

### Lentivirus preparation, transduction, and single cell cloning

Single guide RNAs (sgRNAs) for RSK1 and RSK2 were designed according to the sgRNA sequences in the genome-scale CRISPR knockout library GeCKOv2 (http://genome-engineering.org/gecko/?page_id=15) (Table 1), and oligonucleotides for sgRNA were annealed and cloned into the lentivirus transfer vector lentiCRISPRv2 at the BsmBI restriction site^35^. For the production of recombinant lentiviral particles, the transfer plasmids were co-transfected with the packaging plasmids pMD2.G and psPAX2. Briefly, for each virus, 80% confluent 293FT cells in 6-well plate were transfected in Opti-MEM using 1 µg of the transfer plasmid, 0.5 µg pMD2.G, 0.5 µg psPAX2, and 6 µl of FuGENE HD. After 12 h, the media were replaced with fresh media. After 48 h, the supernatants containing the viral particles were harvested and filtrated through a 0.45 µm-pore membrane. For each viral construct, 1 × 10^6^ U937 cells were transduced in 2 ml of media with 1 ml of viral supernatant in wells of a 6-well plate. At 48 h post-transduction, the media was changed to media containing 2 µg/ml puromycin, and the cells were then further cultured in media with puromycin. For single cell cloning of RSK1- or RSK2-knockout cells, puromycin-selected cells were plated onto a 96-well microplate at a density of 0.5 cells/well and cultured in puromycin-free medium. At 20 days post-plating, the knockout efficiency in 40 single cell clones was examined by western blotting.

**Table 1:** Designed sgRNA guide sequences.

Target	Target site sequence (5′ to 3′)	PAM	Target exon	Strand
RPS6KA1	TTCCAGAATGGACAGACCTC	AGG	2	+
RPS6KA3	ACAGAATGGACAGCAAATTA	TGG	2	−
Neg*	ACGGAGGCTAAGCGTCGCAA			

*Neg, non-targeting sequence.

### Quantitative real-time reverse transcription PCR

U937 cells (2 × 10^6^ cells/ml) were treated with MA_1_ at the indicated concentrations at 37 °C for 1 h. After treatment, total RNA was isolated using TRIzol reagent according to the manufacturer’s instructions. The cDNA was synthesized using the PrimeScript RT reagent kit. Quantitative real-time PCR was performed with SYBR Premix Ex Taq II in a Thermal Cycler Dice Real Time System II (Takara). The relative fold change in mRNA expression was calculated using the ΔΔC_t_ method. *GAPDH* was used as reference gene. Sequences of the primers used were as followed: *PLAU*_for, CAC GCA AGG GGA GAT GAA; *PLAU*_rev, ACA GCA TTT TGG TGG TGA CTT; *GAPDH*_for, CTT CAC CAC CAT GGA GAA GGC; *GAPDH*_rev, GGC ATG GAC TGT GGT CAT GAG.

### Data availability

All data generated or analyzed during this study are included in this published article and its Supplementary Information files.

## Electronic supplementary material

Supporting information

Table S1
