# Supplementary material for: Involvement of RSK1 activation in malformin-enhanced cellular fibrinolytic activity
Source: Sci Rep. 2018 Apr 3;8:5472. doi: 10.1038/s41598-018-23745-0 (PMC5882963; doi:10.1038/s41598-018-23745-0)
Supplement: Supplementary file 1 — Supporting information [file 41598_2018_23745_MOESM1_ESM.pdf]

Involvement of RSK1 activation in malformin-enhanced cellular fibrinolytic activity.

Yukio Koizumi[1,*], Kenichiro Nagai[2], Lina Gao[1], Souichi Koyota[3], Tomokazu Yamaguchi[1], Miyuki Natsui[1], Yumiko Imai[4], Keiji Hasumi[5], Toshihiro Sugiyama[1], Keiji Kuba[1]

[1] Department of Biochemistry and Metabolic Science, Akita University Graduate School of Medicine, 1-1-1 Hondo, Akita 010-8543, Japan

[2] School of Pharmacy, Kitasato University, 5-9-1 Shirokane, Minato-ku, Tokyo 108-8641, Japan

[3] Molecular Medicine Laboratory, Bioscience Education and Research Support Center, Akita University, 1-1-1 Hondo, Akita 010-8543, Japan

[4] Laboratory of Regulation of Intractable Infectious Diseases, National Institute of Biomedical Innovation, Health and Nutrition, 7-6-8 Saito-Asagi, Ibaraki, Osaka 567-0085, Japan

[5] Department of Applied Biological Science, Tokyo Noko University, 3-5-8 Saiwaicho, Fuchu, Tokyo 183-8509, Japan

* Correspondence to: Yukio Koizumi, Ph.D.

Department of Biochemistry and Metabolic Science, Akita University Graduate School of Medicine, 1-1-1 Hondo, Akita 010-8543, Japan. Tel: +81-18-884-6075. Fax: +81-18-884-6443. E-mail: ykoizumi@med.akita-u.ac.jp

Supplementary information

Supplementary Methods

Fluorescence imaging

Sample preparation for fluorescence imaging was performed as described in the main text. Fluorescence 3D image was obtained with a confocal laser scanning microscope LSM510 (Zeiss, Oberkochen, Germany).

Supplementary Figure 1

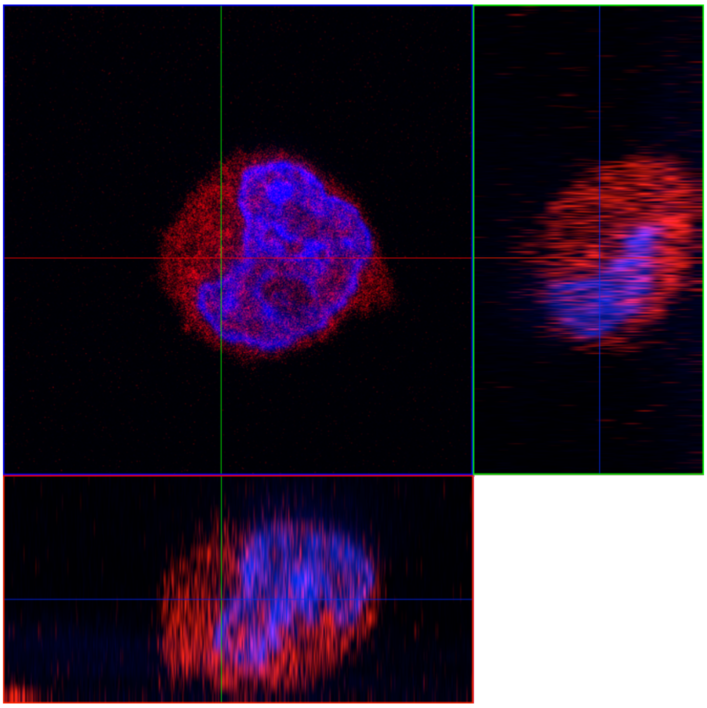

Fig. S1: Fluorescence-conjugated MA1 is localized in cytoplasm.

U937 cells were incubated with MA1-rhodamine (red) in the presence of human platelet-poor plasma on fibrin-coated slide. After fixation and washing, cells were stained with DAPI (blue). Fluorescence 3D image was obtained by a confocal laser scanning microscope.

Supplementary Figure 2

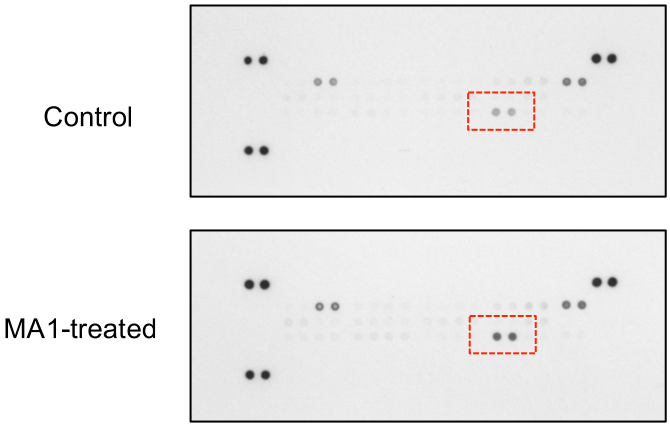

Fig. S2: Uncropped membrane images for Figure 2C.

Supplementary Figure 3

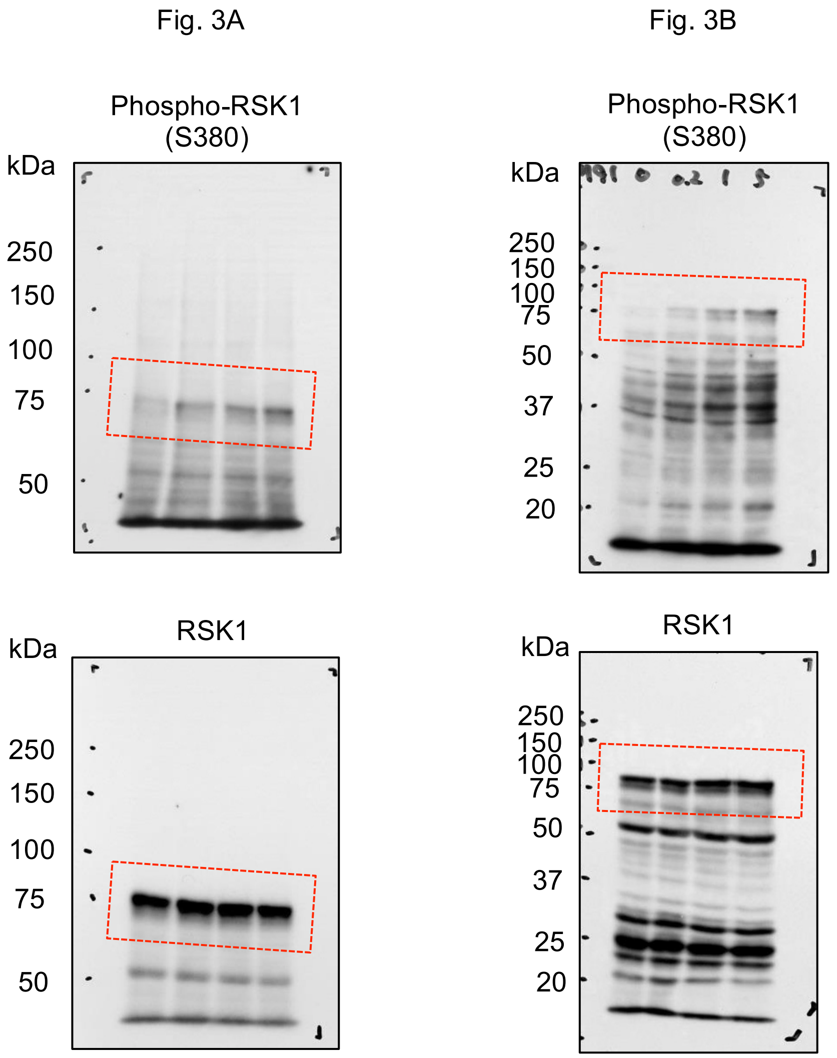

Fig. S3: Uncropped membrane images for Figure 3A and B.

Supplementary Figure 4

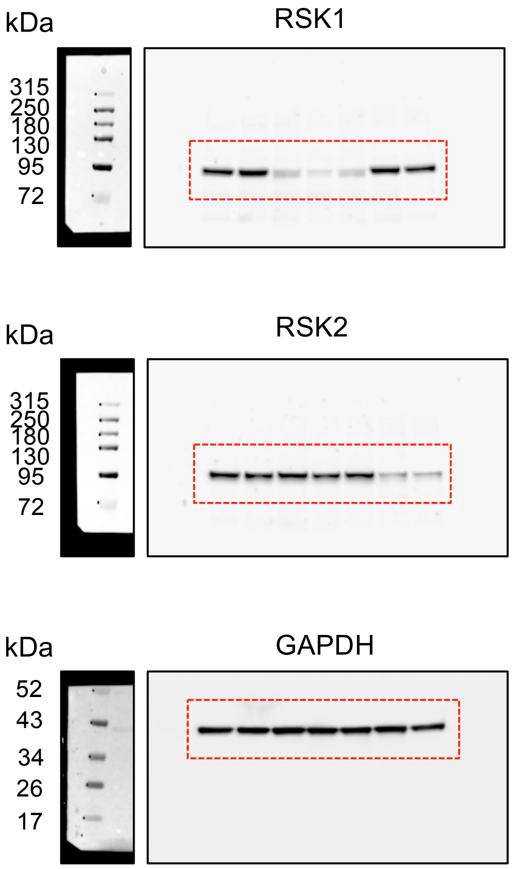

Fig. S4: Uncropped membrane images for Figure 4A.

Supplementary Figure 5

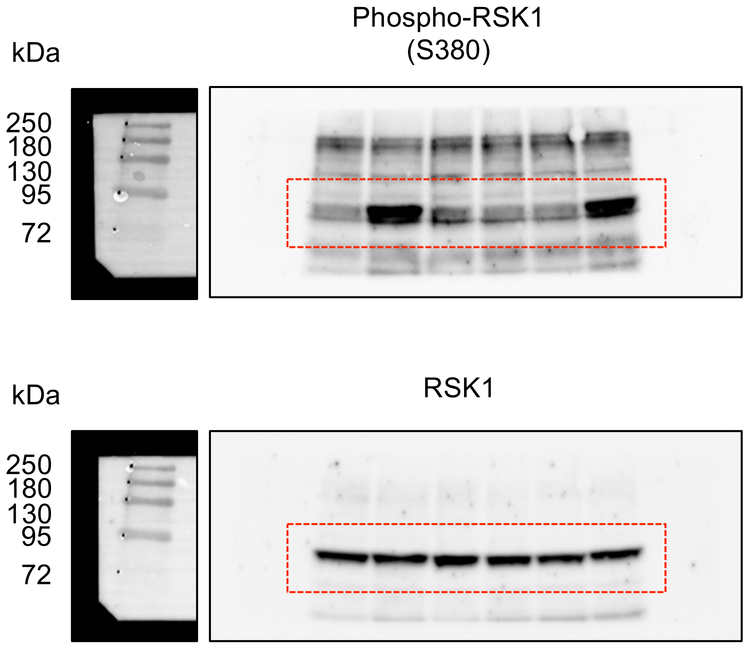

Fig. S5: Uncropped membrane images for Figure 5B.

Supplementary Figure 6

Fig. 6A

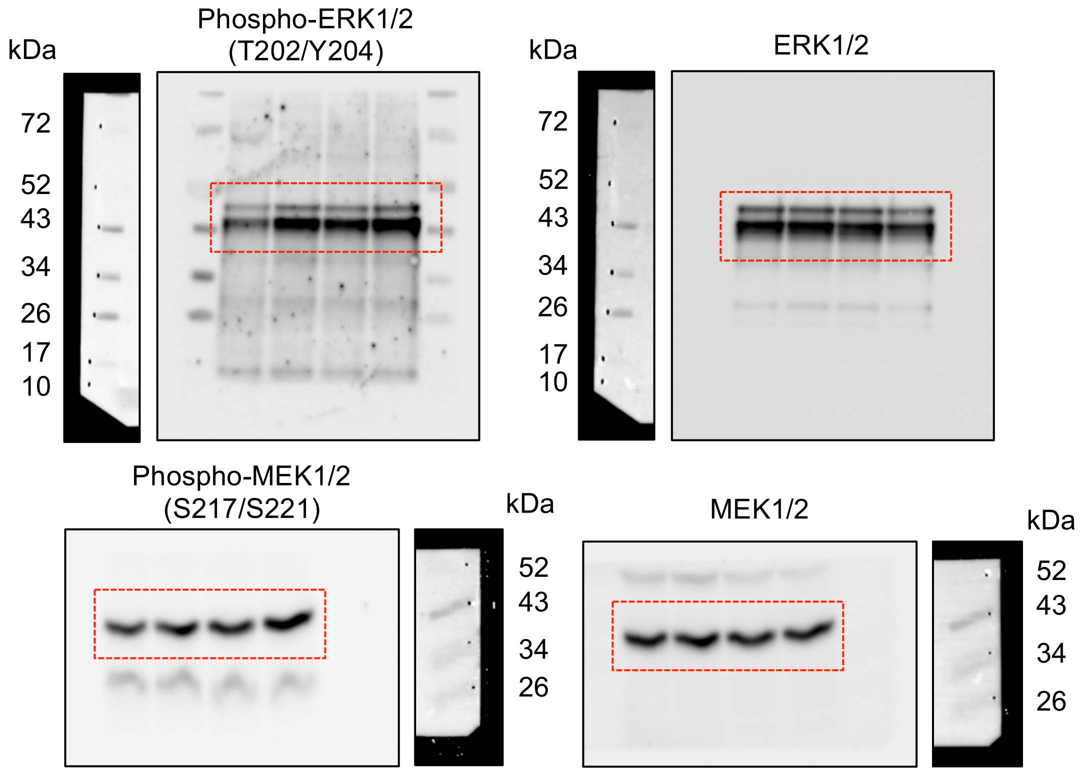

Fig. S6: Uncropped membrane images for Figure 6A.

Supplementary Figure 6, continued

Fig. 6B

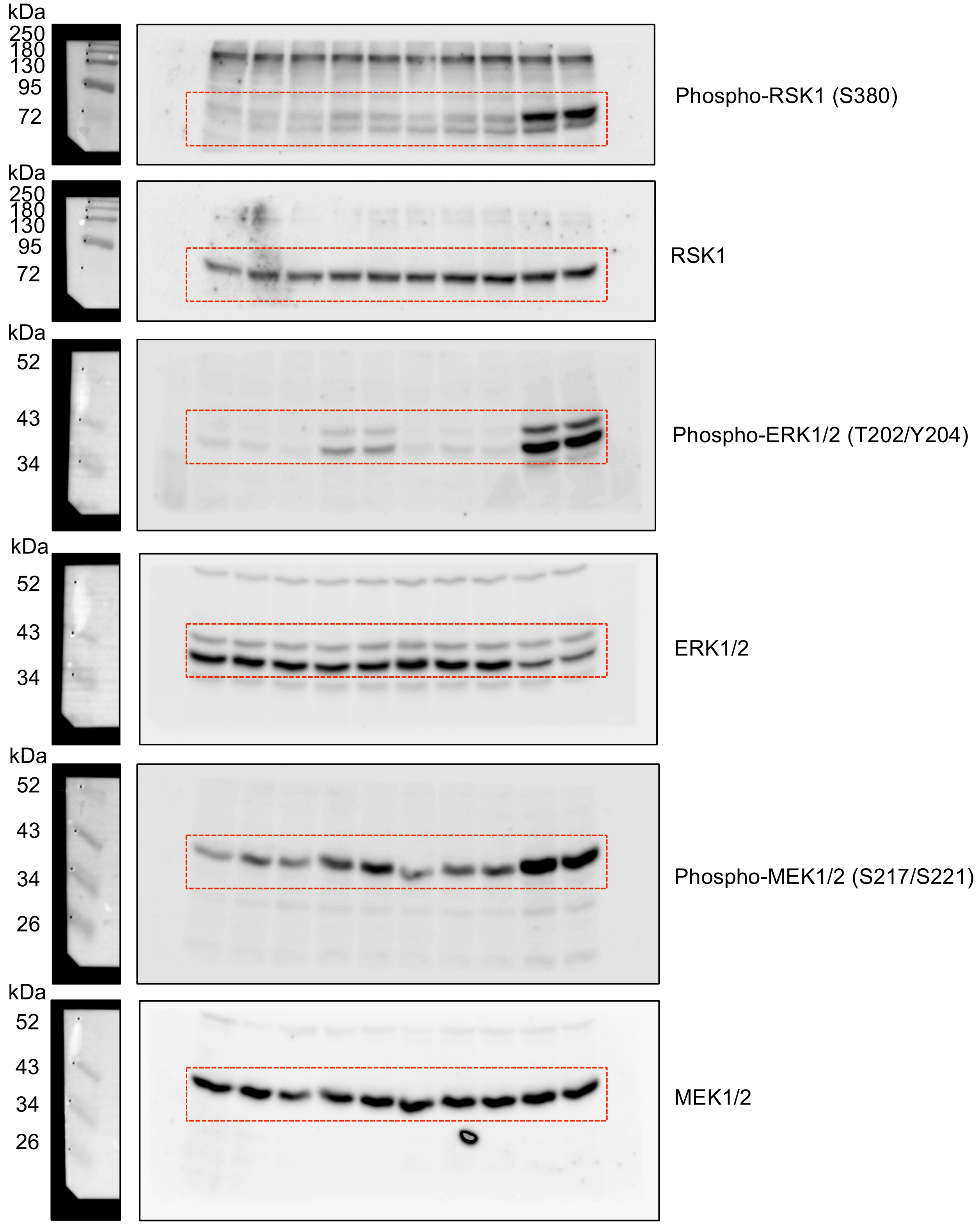

Fig. S6 (*continued*): Uncropped membrane images for Figure 6B.

Fig. 6C

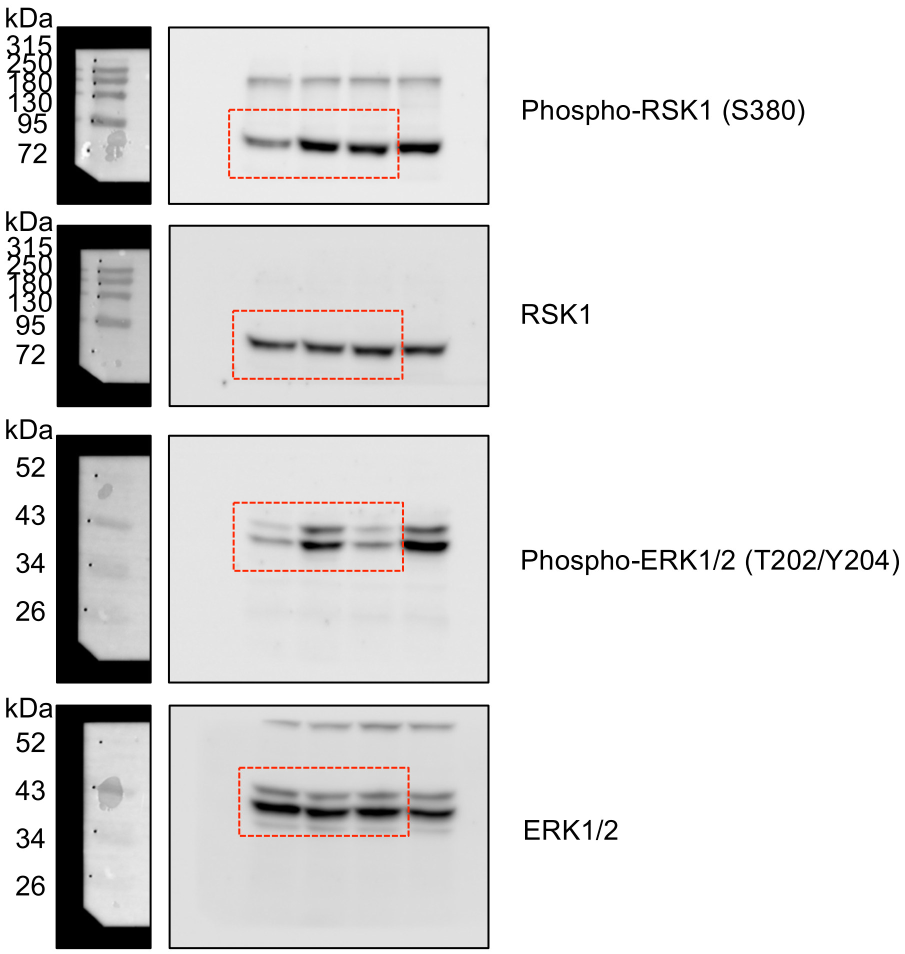

Fig. S6 (*continued*): Uncropped membrane images for Figure 6C.

Fig. 7B

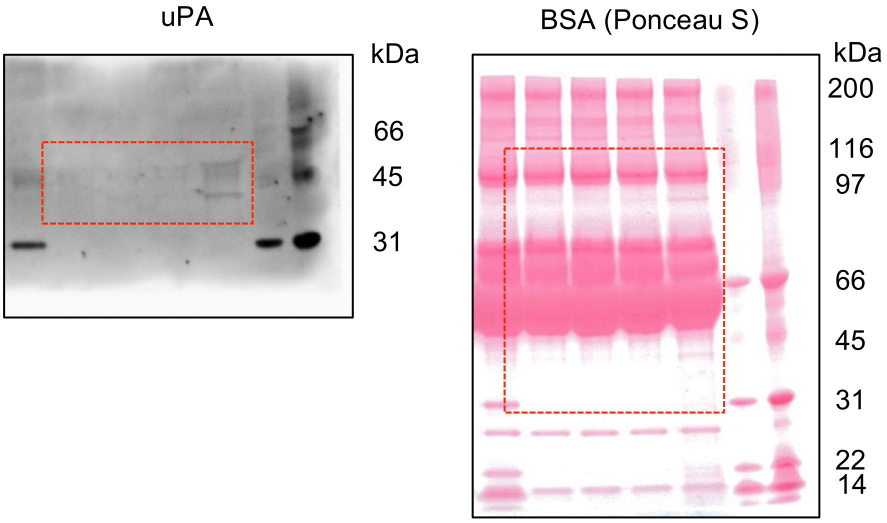

Fig. 7D

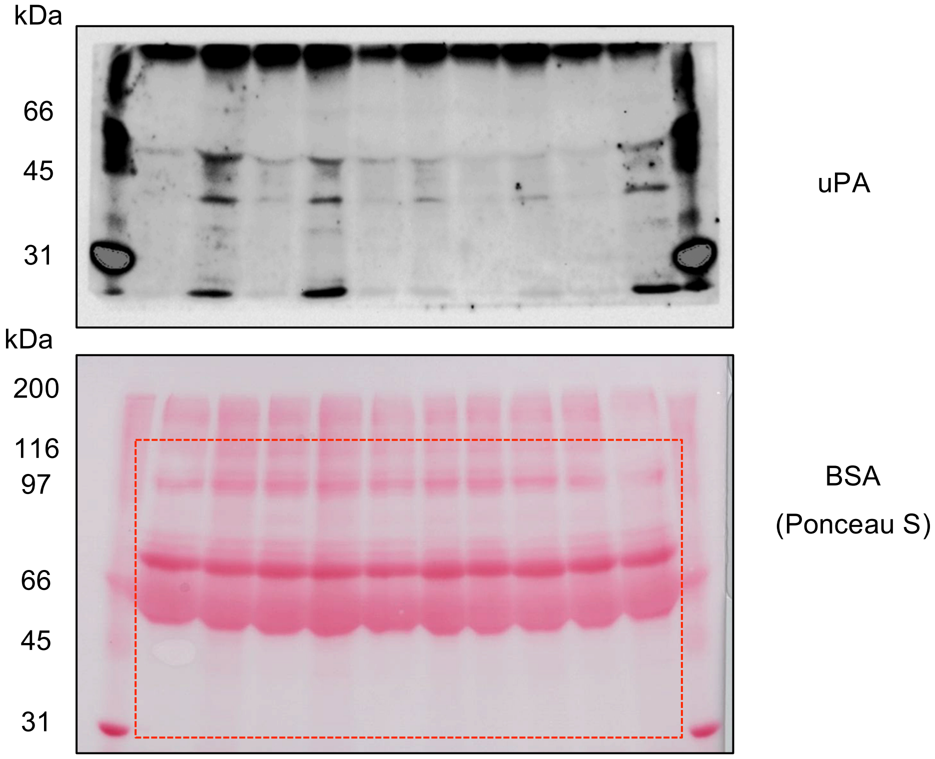

Fig. S7: Uncropped membrane images for Figure 7B and D.
